# Specific DNA duplex formation at an artificial lipid bilayer: fluorescence microscopy after Sybr Green I staining

**DOI:** 10.3762/bjoc.10.240

**Published:** 2014-10-02

**Authors:** Emma Werz, Helmut Rosemeyer

**Affiliations:** 1 Organic Materials Chemistry and Bioorganic Chemistry, Institute of Chemistry of New Materials, University of Osnabrück, Barbarastr. 7, D-49069 Osnabrück, Germany; 2 Ionovation GmbH, Westerbreite 7 (CUT), D-49084 Osnabrück, Germany

**Keywords:** artificial lipid bilayers, lipo-oligonucleotide duplexes, nucleic acids, Sybr Green I

## Abstract

The article describes the immobilization of different probe oligonucleotides (**4**, **7**, **10**) carrying each a racemic mixture of 2,3-bis(hexadecyloxy)propan-1-ol (**1a**) at the 5’-terminus on a stable artificial lipid bilayer composed of 1-palmitoyl-2-oleoyl-*sn*-glycero-3-phosphoethanolamine (POPE) and 1-palmitoyl-2-oleoyl-*sn*-glycero-3-phosphocholine (POPC). The bilayer separates two compartments (*cis/trans* channel) of an optical transparent microfluidic sample carrier with perfusion capabilities. Injection of unlabeled target DNA sequences (**6**, **8**, or **9**), differing in sequence and length, leads in the case of complementarity to the formation of stable DNA duplexes at the bilayer surface. This could be verified by Sybr Green I double strand staining, followed by incubation periods and thorough perfusions, and was visualized by single molecule fluorescence spectroscopy and microscopy. The different bilayer-immobilized complexes consisting of various DNA duplexes and the fluorescent dye were studied with respect to the kinetics of their formation as well as to their stability against perfusion.

## Introduction

The post-biosynthetic lipophilization of various biomolecules such as of proteins and carbohydrates is of decisive importance for the correct function of the cell [1]. With the recent discovery of geranylated tRNAs in bacteria [2] the interest in so-called lipo-oligonucleotides (LONs) has grown tremendously [3–4]. The ability to form complex nano-architectures [5–6] as well as self-assembling aggregates such as micelles, vesicles [3] and bilayer formation of nucleolipids [7] offers numerous possibilities, e.g., for drug delivery [3]. Simultaneously, the interaction of such nanostructures with lipid membranes becomes an ever-greater focus [5–6,8–11].

The study of the interactions of single- and double-stranded nucleic acids with lipid bilayers is, therefore, of significant importance, particularly for the following reasons: 1. for the optimization of the in vivo delivery of lipophilic siRNAs [12–14], 2. for the development of analytical techniques for the detection of nucleic acids [15–17], 3. for structure elucidation of complex aggregates formed by such natural nanostructures [5–6] and 4. for cell-surface engineering [18].

In a preceding manuscript [19] we reported the lipid bilayer immobilization of lipo-oligonucleotides carrying a racemic bis(hexadecyloxy)propan-1-yl tag (**1a**) at the 5’-termini at an artificial lipid bilayer–water phase boundary. These were prepared using the cyanoethyl phosphoramidite **1b**. A specific duplex formation with complementary cyanine-5 (Cy5)-labelled DNA strands (**2b**) – prepared by using compound **2a** – was proven by fluorescence microscopy. Now, we simplify this technique by hybridizing an unlabelled DNA target strand to the bilayer-immobilized lipo-oligonucleotide and by using Sybr Green I (**3**, SG) [20–22] as a fluorescent double strand indicator [20–22]. This bears the advantage that the target DNA – the presence or absence of which is going to be analysed – should not be labelled separately with a fluorochrome tag such as cyanine-5 (Cy5) or TAMRA, neither by chemical synthesis nor by a polymerase chain reaction (PCR).

**Figure 1:**
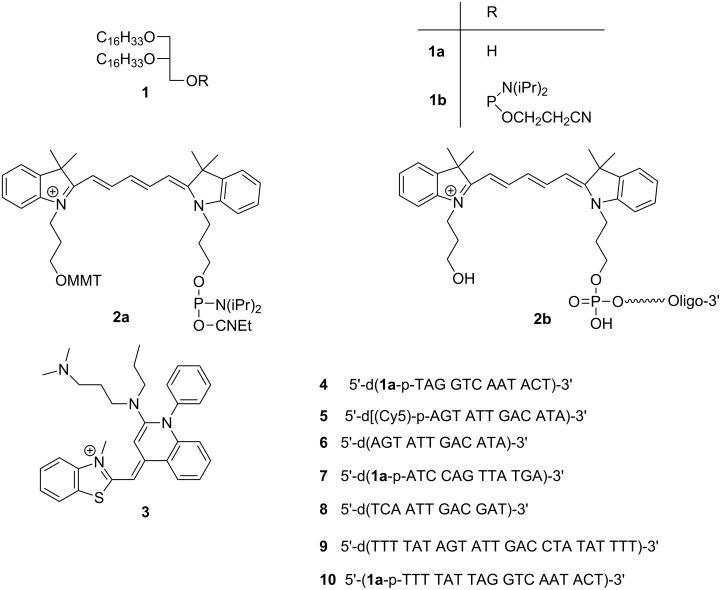
Chemical formulae and lipo-oligonucleotide sequences.

The chemical formulae (**1–3**) as well as the lipo-oligonucleotide sequences the paper is dealing with are shown in Figure 1.

## Results and Discussion

**Figure 2:**
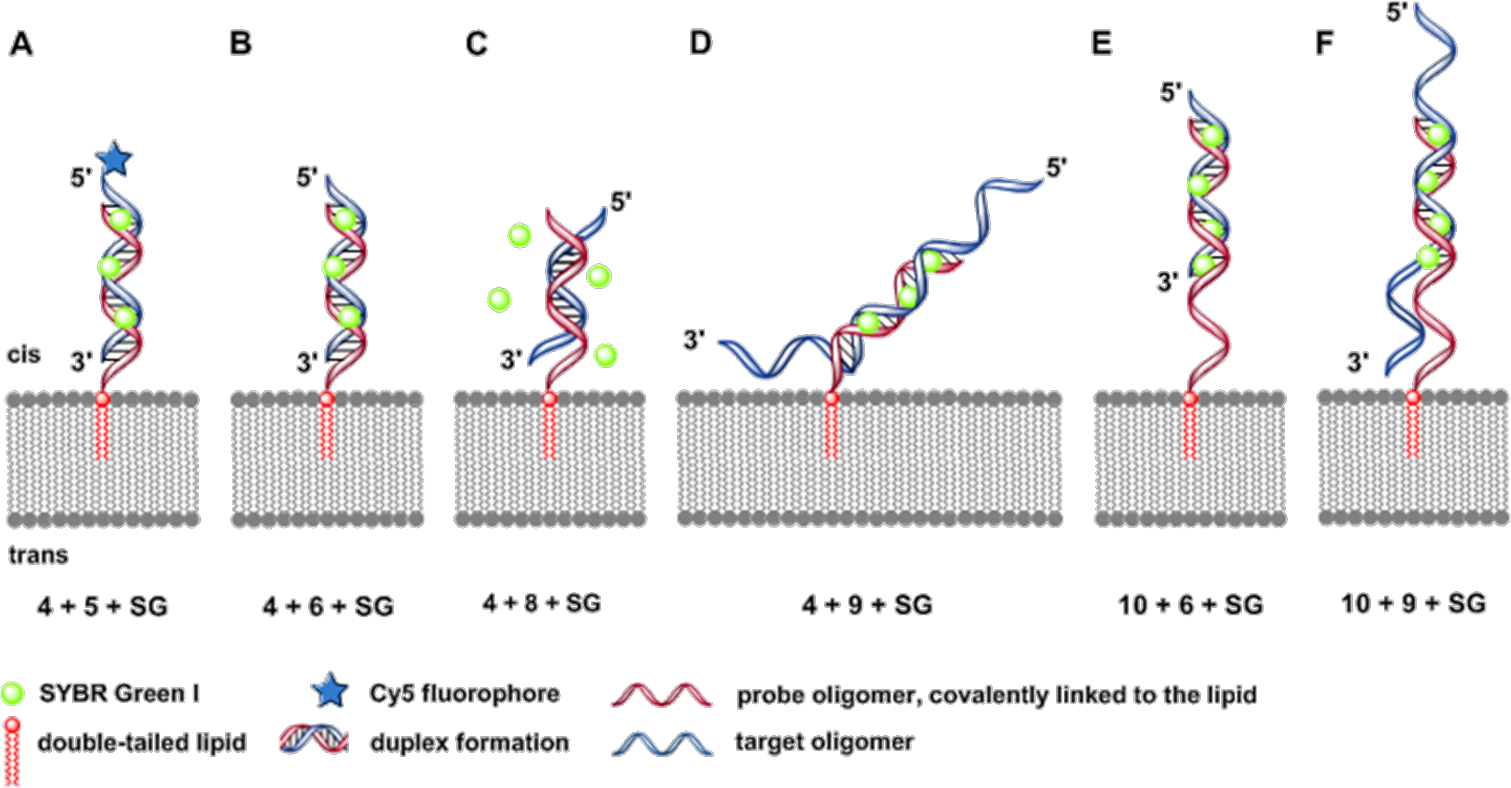
Schematic illustrations (experiments A–F) of the specific DNA duplex formation at artificial lipid bilayers forming structures of various membrane-bound nucleic acid–dye complexes. A) Covalent bound Cy5-fluorophore and intercalated SG with DNA duplex were irradiated simultaneously at 635 nm and 470 nm. B) 100% match of the duplex formation with 12 bp-target oligomer. C) 50% match (6 bp) of the duplex formation with 12 bp-target oligonucleotide. D) 24-mer target oligonucleotide with AT-rich tails, each tail with 6 AT-bases. E) 18-mer probe oligonucleotide with a free AT-rich tail of 6 bases as a spacer covalently linked to the double-tailed lipid, forming a 12 bps duplex with the target sequence. F) The same lipo-oligonucleotide as described in (E) forms a 12 bps duplex in combination with a target oligonucleotide, resulting in free overhanging AT-rich tails at the 5’- as well as 3’-ends, each tail containing 6 nucleobases.

Figure 2 displays the six DNA duplexes which have been assembled at the artificial lipid bilayer and visualized by addition of Sybr Green I.

**Figure 3:**
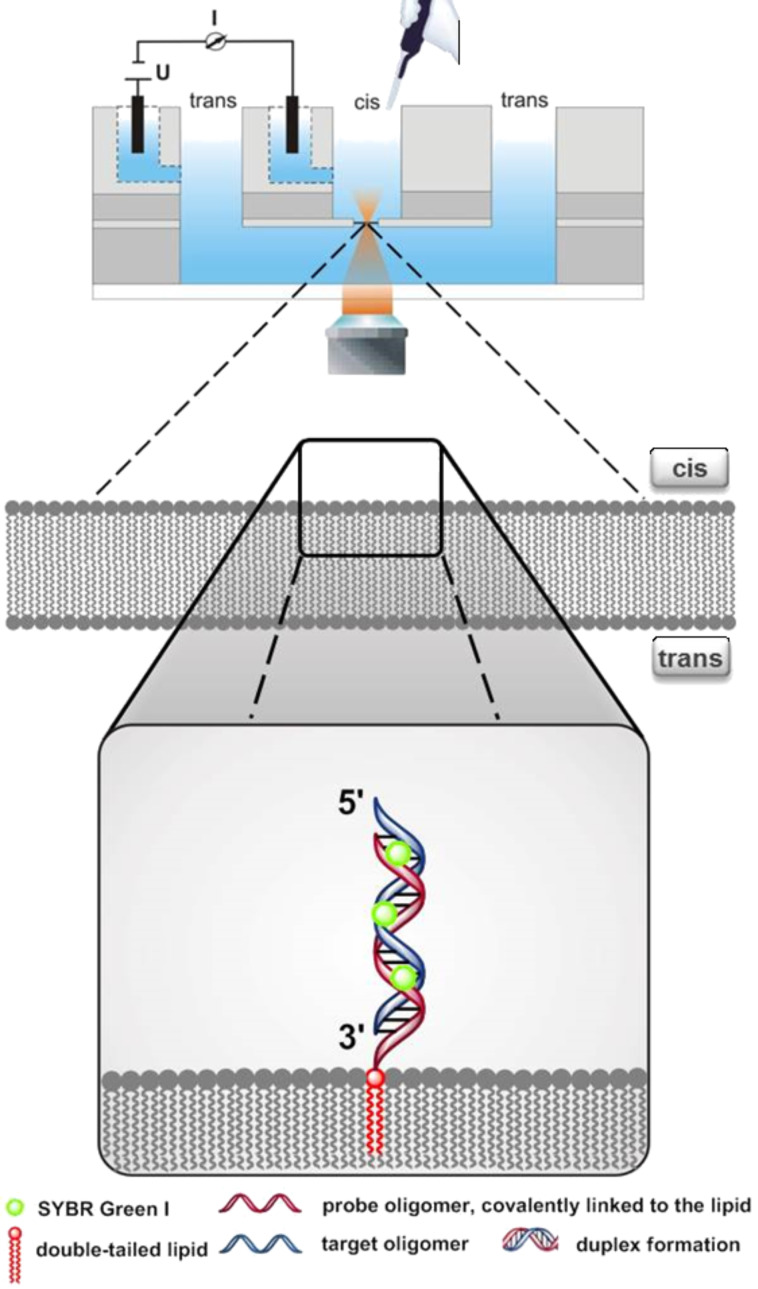
Experimental setup. Schematic drawing of the laser scanning microscope, the optical transparent microfluidic bilayer slide, and the lipid bilayer with the incorporated double-tailed lipo-oligonucleotides as well as of Sybr Green I, forming a membrane - bound nucleic acid duplex–dye complex.

In the following the results of these experiments regarding the kinetics of incorporation into the bilayer (or its formation) as well as the release of the ternary complex (or its disaggregation) upon perfusion of the *cis* channel is described. Figure 3 illustrates a scheme of the experimental setup (for details see Experimental).

Experiment A: **4** + **5** + **SG**. In a preceeding manuscript we have studied the bilayer incorporation of the lipo-oligonucleotide **4** (5’-d(**1a**-TAG GTC AAT ACT)-3’) and its successful and specific duplex formation with the cyanine-5-labeled complementary strand **5** (5’-d[(Cy5)-p-AGT ATT GAC CTA]-3’) [19]. As a negative control it had been shown that a membrane-bound duplex formation between the lipo-oligonucleotide **7** (5’-d(**1a**-ATC CAG TTA TGA)-3’) and the oligomer **5** failed. Now, we repeated the first experiment (**4** + **5**) successfully and run a z-scan of the bilayer after 45 min of incubation with a laser irradiation of the Cy5 dye at 635 nm (Figure 2).

**Figure 4:**
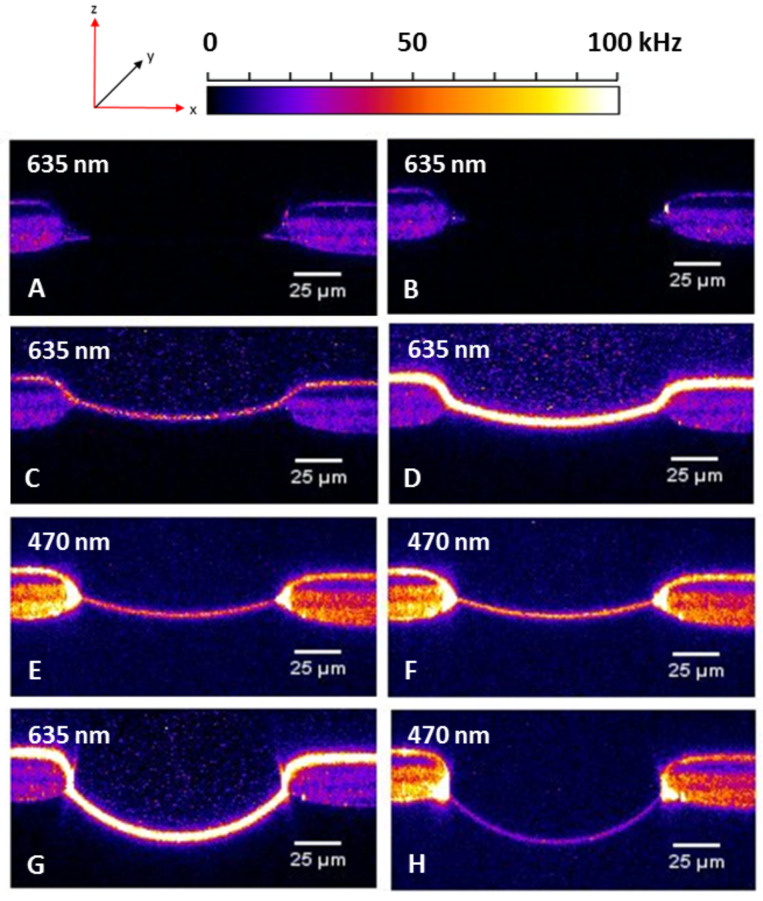
Chronological protocol of duplex formation of the lipo-oligonucleotide **4** with the complementary Cy5-labelled target strand **5** with simultaneous staining of the DNA duplex with Sybr Green I. A) Z-Scan of an empty bilayer; after the bilayer has been formed, both compartments (*cis* and *trans*) were perfused for 30 s (1.1 mL/min, each). Cy5 irradiation at 635 nm. The z-scan as in (A) with a Sybr Green I irradiation at 470 nm is not shown. As demonstrated in (A) there is no fluorescence in the bilayer. B) Z-Scan after addition of the oligomer **4** (8 µL, 500 nM) to the *cis* compartment, followed by 45 min of incubation, Cy5 irradiation at 635 nm. C) Z-Scan after addition of the oligomer **5** (8 µL, 50 nM) to the *cis* compartment and 45 min of incubation; Cy5 irradiation at 635 nm. D) Z-Scan after addition of Sybr Green I (8 µL), followed by 45 min of incubation; Cy5 irradiation at 635 nm. E) Z-Scan as in (D), but with Sybr Green I irradiation at 470 nm. F) Z-Scan after an incubation of 5 min and Sybr Green I irradiation at 470 nm. G) Z-Scan after 30 s of perfusion of the *cis* compartment (1.1 mL/min) and Sybr Green I irradiation at 635 nm. H) Z-Scan as in (G), but with Sybr Green I irradiation at 470 nm.

Then, a Sybr Green I (**3**) solution in dimethyl sulfoxide (≈1 µg/mL) was added to the *cis* compartment of the slide. In the first experiment (A) laser irradiation of the intercalated dye was performed at 470 nm. In orienting experiments Sybr Green I was irradiated at 470 nm, in order to see if this dye stains the complementary DNA strands **4** + **5**, which are known to form a duplex at the bilayer membrane [19]. Firstly, we evaluated the optimal measuring conditions such as the volume, the Sybr Green I concentration as well as of the lipo-oligonucleotides. At this point we first wanted to find out if a non-optimal wavelength of 470 nm would be sufficient for an efficient irradiation of the dye. It turned out, however, that an irradiation of Sybr Green I needs an irradiation wavelength which meets the optimal absorption wavelength to a higher extent. In subsequently performed experiments (B to F) the intercalated Sybr Green I was irradiated at 488 nm because this wavelength is close to its absorption maximum at 494 nm [20–22]. The experiments confirmed a duplex formation in an antiparallel mode on the bilayer surface being stable towards perfusion (Figure 4).

**Figure 5:**
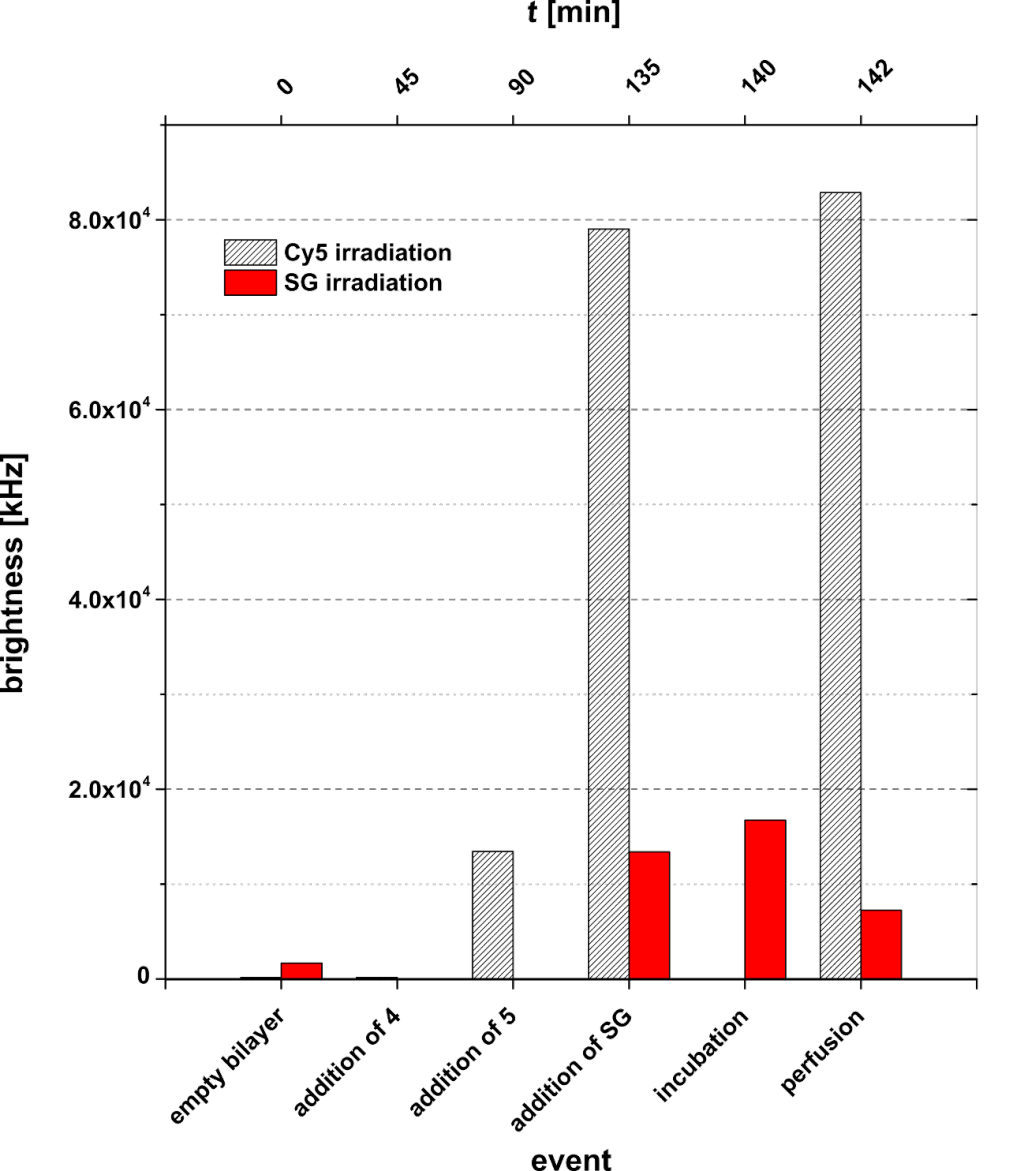
Comparison of the bilayer brightness intensity with either Cy5 (irradiation: 635 nm) or Sybr Green I staining (irradiation: 470 nm) of the **4** + **5** + **SG** complex.

Figure 5 demonstrates the difference in the bilayer brightness upon irradiation of either cyanine-5 (635 nm) [19] or of the ternary complex with SG (**4** + **5** + **SG**) (470 nm). For the determination of the bilayer brightness a region-of-interest (ROI) was defined around the bilayer, and subsequently the density of the intensity counts were summarized (for details, see Experimental).

**Figure 6:**
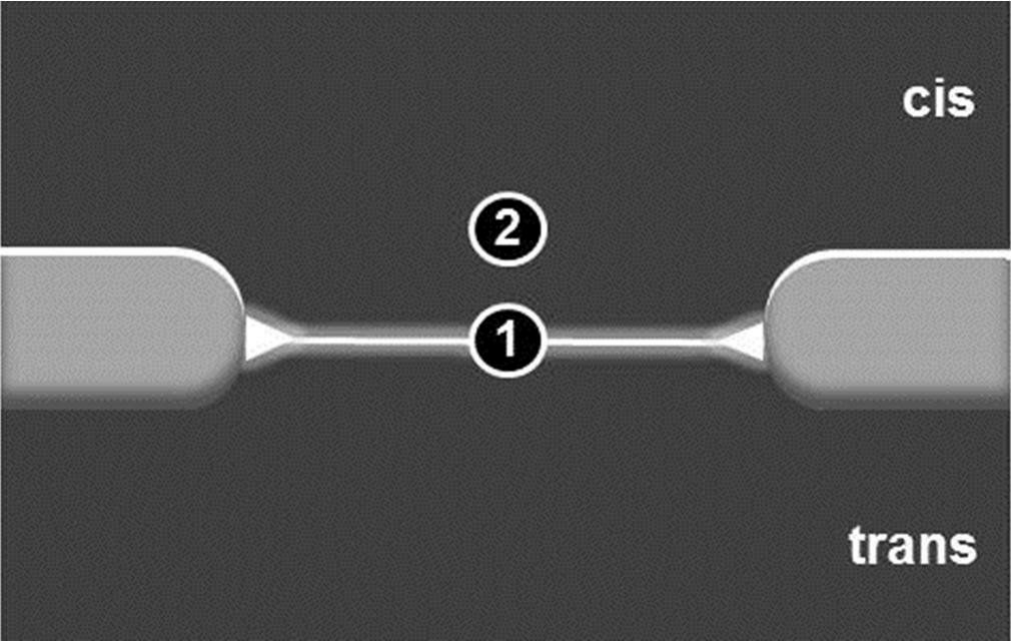
Scheme of a z-scan of a lipid bilayer showing two locations for measurements of the diffusion times. Location 1: bilayer; location 2: solution in close proximity to the bilayer surface.

Besides the bilayer brightness, also the diffusion time, *t*_D_, of the lipo-oligonucleotide duplex–Sybr Green I complex (i) within the bilayer (location 1) and (ii) above the bilayer (location 2) was determined (Figure 6).

Table 1 and Table 2 summarize the diffusion times [*t*_D_ (ms)] of the complexes **4·5·SG** and **4·6·SG** before and after a perfusion at the two locations 1 and 2 as illustrated in Figure 6. It can be clearly seen that the diffusion time of the aggregates is significantly longer within the bilayer compared to the region slightly above (location 2), proving the strong immobilization of the lipophilized DNA duplex within the bilayer via the lipophilic head group. The fastest diffusion of the aggregates occurs in free solution without bilayer (Table 2) [19].

**Table 1:** Diffusion times [*t*_D_ (ms)] of the **4·5·SG** complex in the presence of a lipid bilayer, either by irradiation of the cyanine-5 dye or by irradiation of intercalated Sybr Green I within the **4·5·SG** complex, before and after perfusion of the *cis* compartment. Location 1: bilayer; location 2: solution in close proximity to the bilayer (Figure 6).

	sample			
	**4**·**5**·**SG** complex			
fluorescence signal	Cy5 – irradiation_(635 nm)_		**SG** – irradiation_(470 nm)_	

*t*_D_ at location 1 [ms]	before perfusion	after perfusion	before perfusion	after perfusion
	17.23 ± 2.0^a^	10.33 ± 2.0	0.60 ± 0.09	1.20 ± 0.36
*t*_D_ at location 2 [ms]	2.47 ± 0.3^a^	1.28 ± 0.05	0.72 ± 0.11	0.13 ± 0.05

^a^Data were taken from [19].

**Table 2:** Diffusion times [*t*_D_ (ms)] of the **4·6·SG** complex in the presence of a lipid bilayer in free solution without bilayer as well as in the presence of a bilayer after a 1. and 2. perfusion of the *cis* compartment. Location 1: bilayer; location 2: solution in close proximity to the bilayer (Figure 6).

	sample	
	**4**·**6**·**SG** complex	

*t*_D_ in solution without bilayer [ms]	0.03 ± 0.002	
*t*_D_ at location 1 [ms]	perfusion	
	1.	2.
	1.30 ± 0.28	1.56 ± 0.22
*t*_D_ at location 2 [ms]	0.48 ± 0.71	0.07 ± 0.03

**Figure 7:**
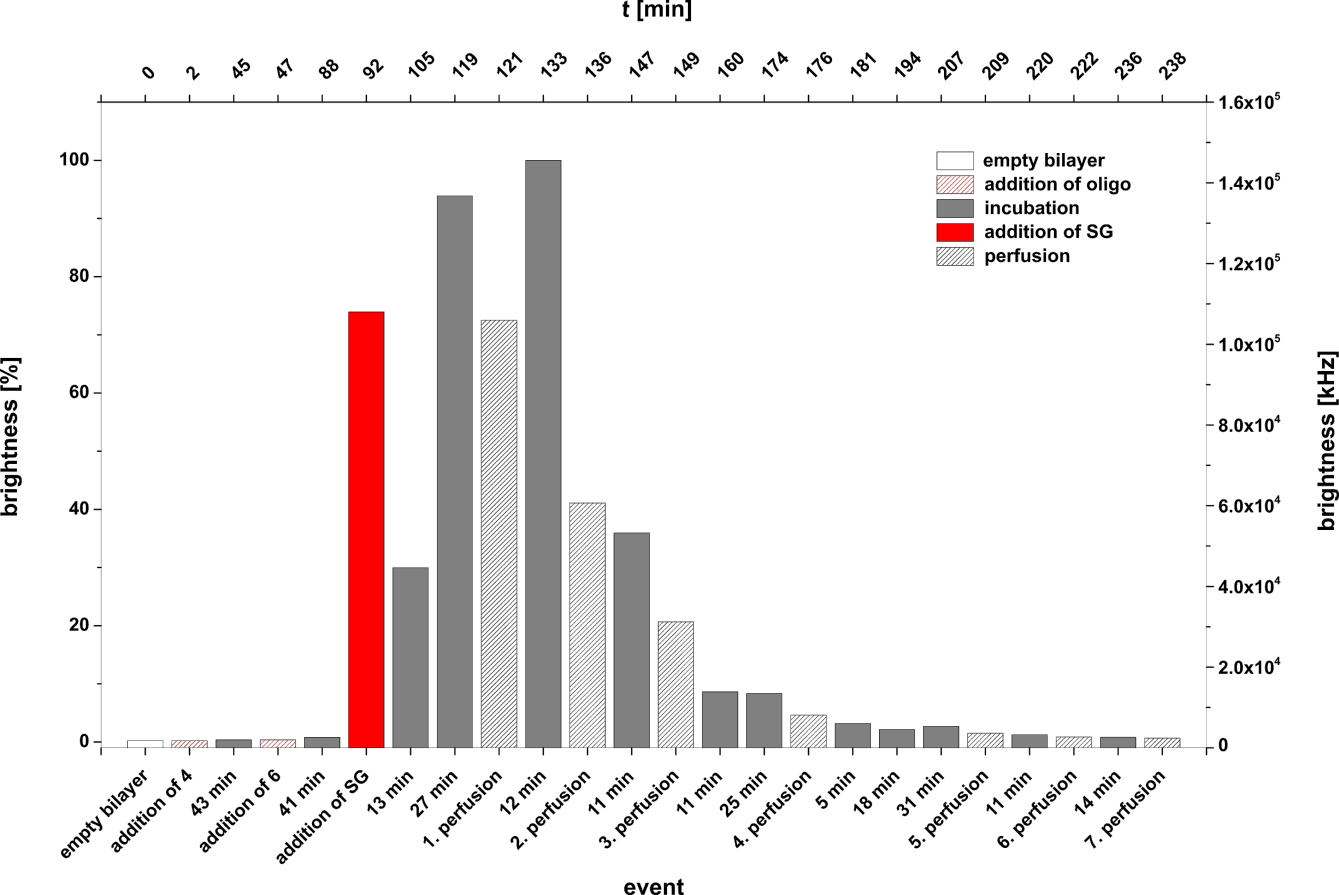
**Experiment B (4** + **6** + **SG)**. Bilayer brightness as function of the various events (addition of oligonucleotides, addition of Sybr Green I, perfusion number as well as of time [min]) within one graph.

Experiment B: **4** + **6** + **SG** and control experiment: **7** + **6** + **SG***.* In a second series of experiments we used an unlabelled target DNA (**6**) for duplex formation at the lipid bilayer and added only a DMSO solution of Sybr Green I to the *cis* compartment of the bilayer slide. As can be seen from Figure 7 also the hybridization of an unlabelled target oligomer **6** with the lipo-oligonucleotide **4** can be proved by adding the dye and irradiation at 488 nm. In case of the incorporation of the lipo-oligonucleotide **7** into the bilayer and addition of the non-complementary unlabelled oligomer **6** (negative control experiment), no duplex formation occurs (data not shown).

From Figure 7 it can be seen that a stepwise mixing of the lipo-oligonucleotide **4** and the complementary strand **6** with an intermediary incubation of 43 min for an optimal insertion of **4** into the bilayer, followed by a further waiting period (41 min) for duplex formation leads to a bilayer brightness of 70% upon addition of Sybr Green I. Two subsequent incubation periods of totally 40 min enhances the normalized brightness of 100% (≈15.2 × 10^4^ kHz). In the following 7 perfusion steps (see Experimental), interrupted by several incubation periods over a period of about 2 h, led to an almost total decrease of the brightness dropping to about 5%. This decrease might be due to either a release of the dye from the intact duplex or to a full disintegration of the ternary complex (**4** + **6** + **SG**).

Experiment C: **4** + **8** + **SG**. In this experiment the lipo-oligonucleotide **4** is inserted into the lipid bilayer, followed by a dodecamer (**8**) which matches the sequence of **4** only in its innermost part to 50%. An addition of Sybr Green I does not lead to a fluorescent bilayer indicating an unstable ternary complex at room temperature. This is in contrast to the experiment B where a 100% match of both dodecamers exists.

Experiment D: **4** + **9** + **SG**. In a further series of experiments we studied the duplex formation of the lipo-oligonucleotide **4** and the oligomer **9** at the lipid bilayer–water phase boundary layer. The oligomer **9** matches **4** to 100% within its innermost part but contains hexameric overhangs at both termini. Duplex formation was again indicated using Sybr Green I.

**Figure 8:**
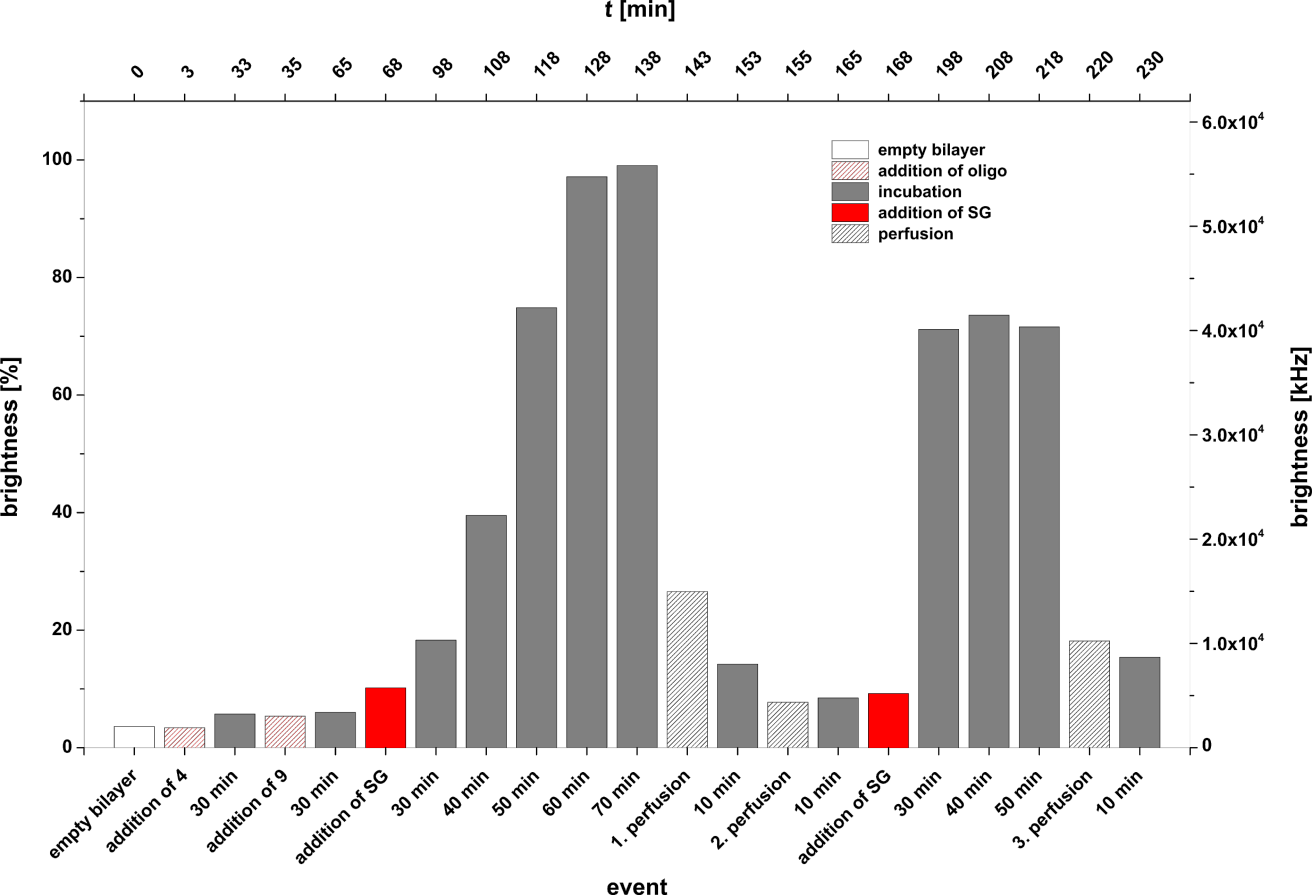
Experiment D (**4** + **9** + **SG**). Bilayer brightness as function of time and the various events [addition of **4** (at *t* = 3 min); addition of **9** (at *t* = 35 min)**,** addition of Sybr Green I (at *t* = 68 min), incubation periods, perfusions].

In this case an interesting phenomenon was observed: after immobilization of oligomer **4** within the bilayer and after addition of the complementary strand **9** 30 min later as well as of Sybr Green I, further 30 min later, a full development of maximal fluorescence (normalized maximal brightness of 100% ≈ 5.6 × 10^4^ kHz) of the bilayer could only be observed after ≈1 h (Figure 8). This time of formation of the ternary complex is significantly slower than in the experiments with blunt-ended duplex formation reactions described before, which show an almost spontaneous complex formation with the intercalating dye. Moreover, the complex between **4** and **9** as well as of Sybr Green I at the bilayer seems to be highly labile because already a single perfusion step of the *cis* compartment (1 min, 1.1 mL of buffer at *t* = 143 min) leads to an almost 90% disappearance of the fluorescence. Interestingly, however, is the finding that a renewed addition of a Sybr Green I solution to the *cis* compartment (at *t* = 168 min) leads again to the appearance of fluorescence 40 min later (brightness, 70%), indicating a partial reconstruction of the complex consisting of **4**, **9**, and the intercalating dye.

**Figure 9:**
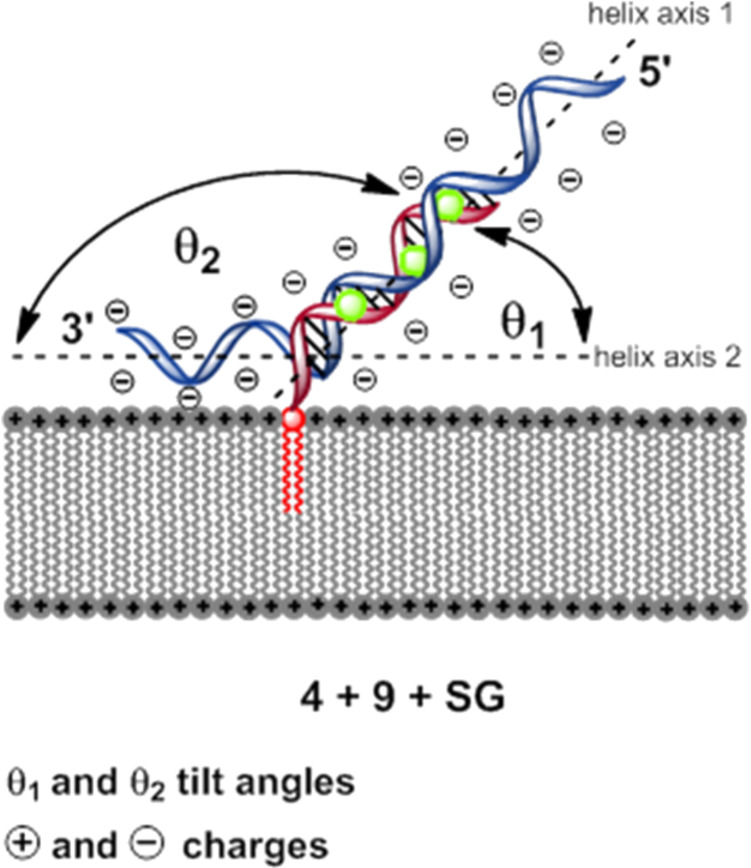
Conceivable geometry of the complex at the bilayer surface (*cis* compartment).

The results described above offer several possibilities of interpretation: The target strand **9** carries 3’-terminally to the recognition site of the lipo-oligonucleotide **4** an overhang of 6 nucleotides in length. Therefore, duplex formation between **4** and **9** over a full length of 12 base pairs leads inevitably to a clash of the overhang with the bilayer surface. As the head groups of the neighbouring bilayer molecules (POPC, POPE) are both positively charged and the internucleotide residues of the overhanging oligonucleotide are negatively charged, a strong Coulomb attraction should exist which can only be shielded by solvation of the lipid head groups and of the nucleic acid phosphodiester groups as well as by the metal cations of the surrounding buffer. The supramolecular assembly of all reaction partners might look like as shown in Figure 9. From this figure it can be deduced that two different tilt angles (θ_1_ and θ_2_) probably exit between the bilayer surface and the double helical part (θ_1_) as well as between the single stranded sequence and the surface (θ_2_). A stretched-out, parallel association of the full length nucleic acid with the bilayer surface can be most probably ruled out. In such a case a conformational constraint would arise between the lipid head group and the appending oligonucleotide. Moreover, a Cy-5-labelled oligonucleotide such as compound **5** should be bound tightly to the bilayer surface leading to a strong and stable fluorescence of the bilayer which could not be observed.

It is, however, obvious that a severe disturbance of either the complex formation between the probe lipo-oligonucleotide **4** and the target nucleic acid **9** and/or the intercalation of the Sybr Green dye by the overhang take place. This might be the reason for the instability of the assembled complex at the lipid bilayer.

Experiment E: **10** + **6** + **SG** as well as in a reversed order of addition (**SG** + **10** + **6**). Next, the oligomer **10** was prepared which contains the lipophilic 5’ head group **1a** and the recognition sequence separated by an oligonucleotide spacer having the same length as the target sequence overhang (6 bp).

**Figure 10:**
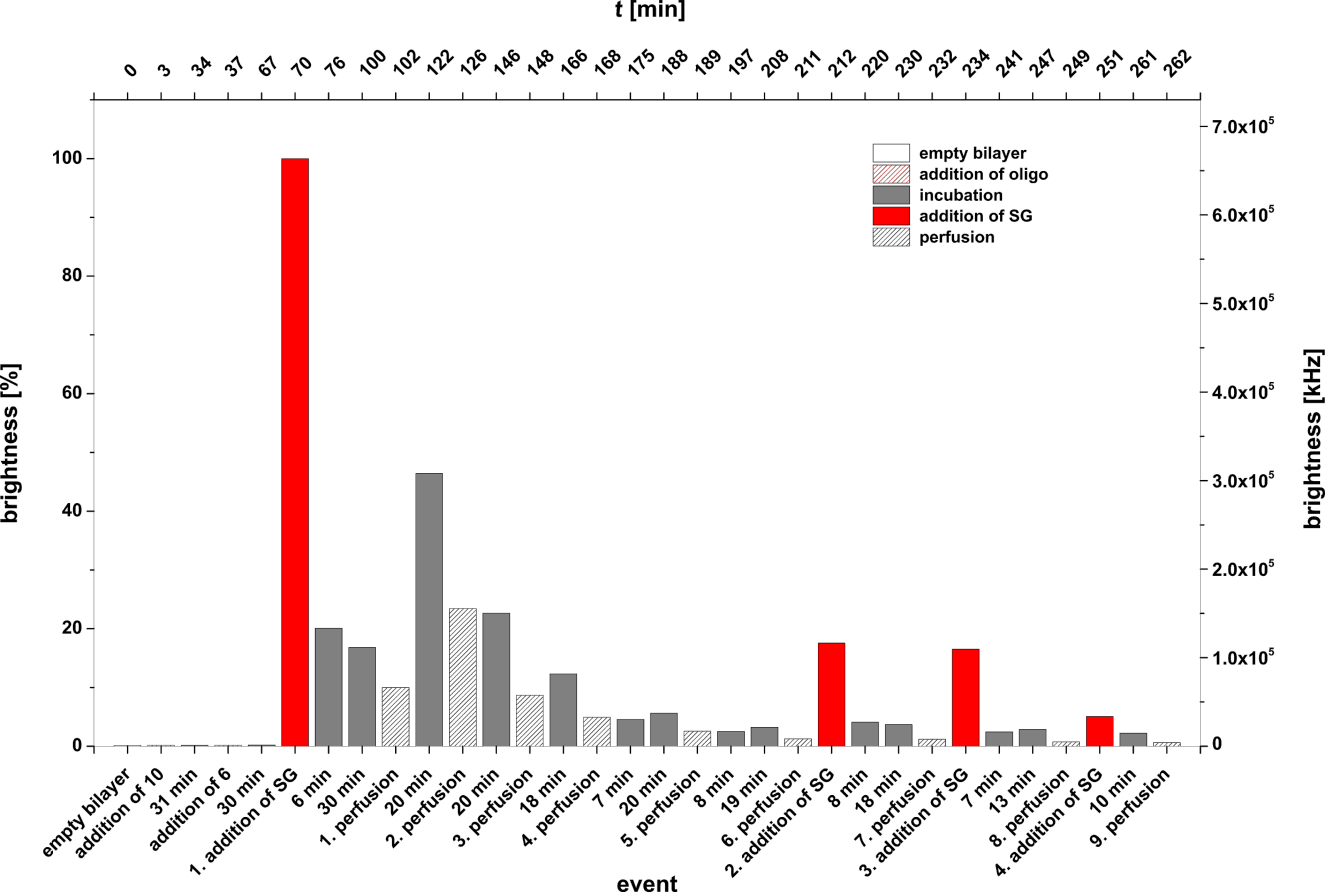
Experiment E (**10** + **6** + **SG**). Bilayer brightness as a function of time as well as of various events [addition of the lipo-oligonucleotide **10** (at *t* = 3 min); addition of the oligomer **6** (at *t* = 37 min); addition of Sybr Green I (at = 70 min, 212 min, 234 min, and 251 min); incubation periods, perfusions].

**Figure 11:**
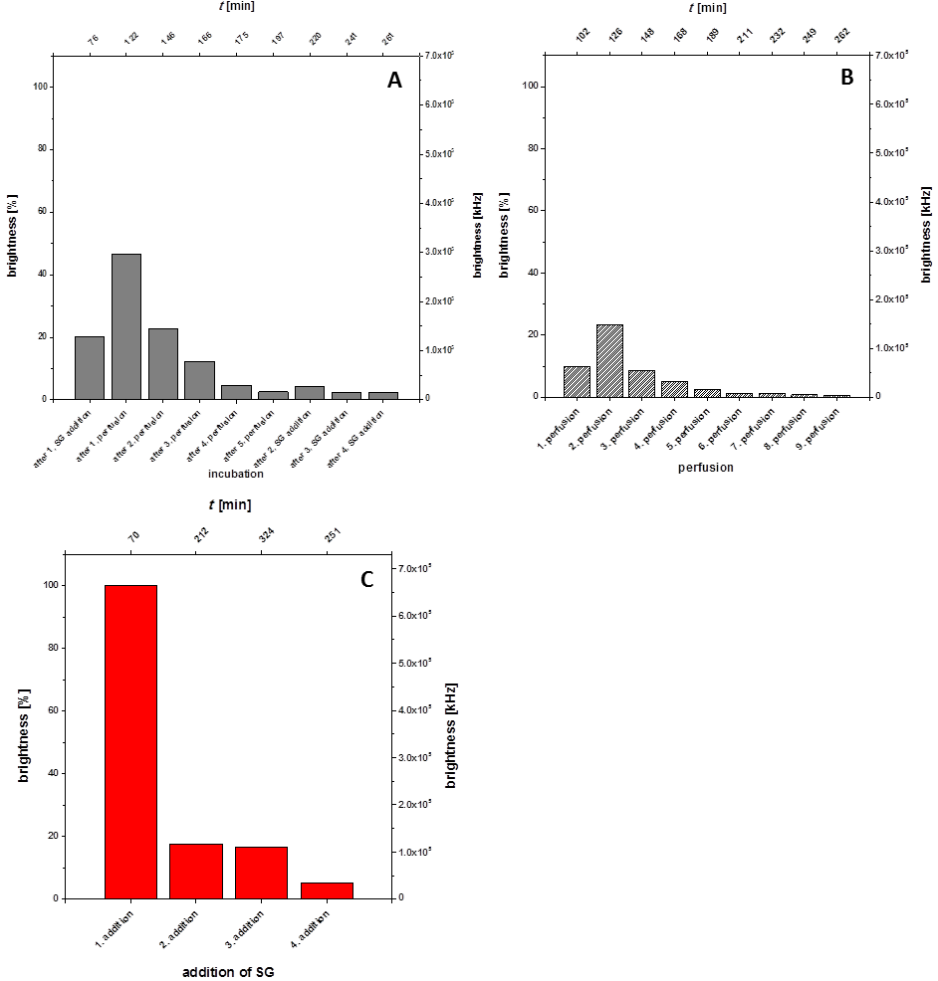
Experiment E (**10** + **6** + **SG**). Bilayer brightness as function of the incubation time (A), of the perfusion number (B) and of Sybr Green I addition (C).

From Figure 10 it can be seen that in this case the ternary complex formation occurs spontaneously upon addition of a first portion of the dye solution. This is, however, not a stable situation because an incubation of only 6 min (without perfusion!) leads to a reduction of the bilayer brightness of just 20%. Several further waiting periods, interrupted by 6 perfusions, reduced the bilayer brightness from the original 100% (100% normalized, ≈6.6 × 10^5^ kHz) to less than 5% within 2.5 h. A further addition of Sybr Green I at *t* = 212 min, however, enhances the bilayer brightness again to about 20%, indicating the presence of a substantial amount of the intact DNA duplex at the lipid bilayer surface on the *cis* side. Subsequent incubation periods, followed by single perfusion steps reduced the bilayer brightness each time to about 5%. A repeated Sybr Green I addition at *t* = 234 min leads again to an enhancement of the brightness value of 18%. A 4^th^ addition of Sybr Green I at 251 min results in an only very slight brightness enhancement of ≈5% (Figure 11).

**Figure 12:**
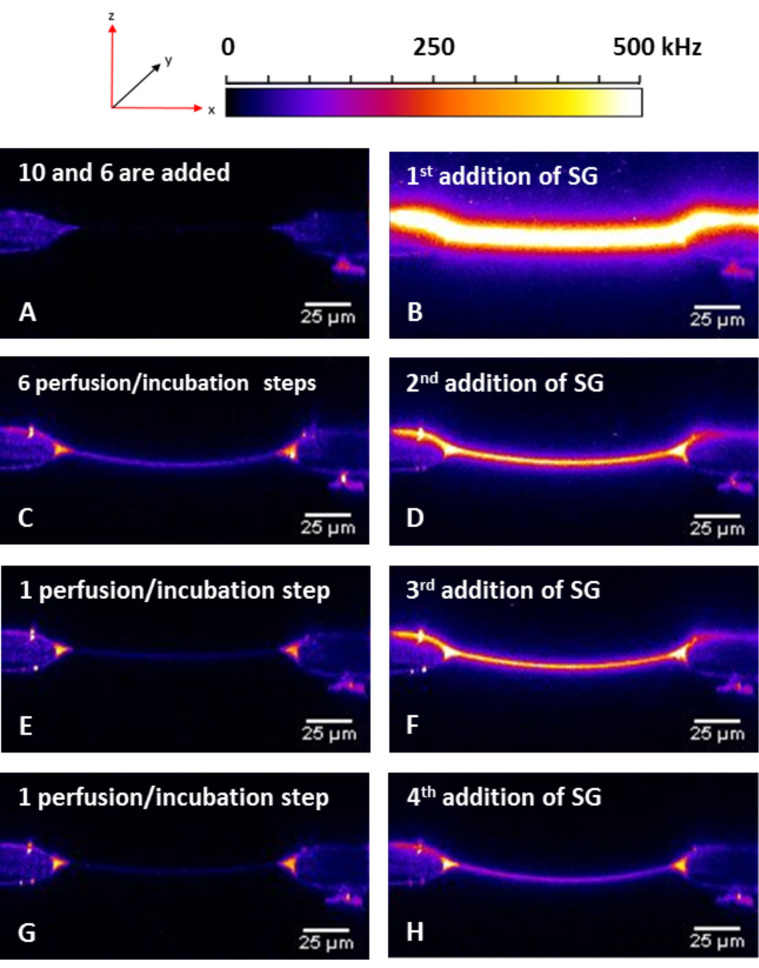
Z-scans of the experiment E before and after the addition of Sybr Green I. A) Z-scan after the addition and incubation of the lipo-oligonucleotide **10** and of the complementary target strand **6**, respectively. There is no brightness of the bilayer. B) Z-scan quite after the first addition of Sybr Green I. The brightness rises immediately because of the DNA duplex formation of the lipo-oligonucleotide **10** and of the complementary target strand **6**. C) Z-Scan after six perfusions each followed by an incubation step resulting in an decreasing of the brightness in the bilayer. D) Second addition of SG I leads to an increase of the brightness. E) Only one perfusion/incubation step removes the brightness of the bilayer completely. F) The third addition of Sybr Green I leads to an increase of the brightness in the bilayer on the same level as it was reached after the second SG addition. G) After a further perfusion/incubation step the brightness of the bilayer is removed again, H) but it does not rises as high as shown before.

In Figure 12 the z-scans of the Experiment E – before and directly after Sybr Green I additions – are displayed. As already shown in Figure 10 and Figure 11 the bilayer brightness increases with each Sybr Green I addition. After 6 perfusions and incubation steps the brightness is reduced to almost zero (*t* = 211 min), however, with the next additions of the dye the brightness increases again. Only after seven perfusion and incubation periods the brightness does no longer increase significantly, which might be traced back to the fact that the immobilized DNA duplex is meanwhile dissociated, and that the non-lipophilized strand is has been washed out.

**Figure 13:**
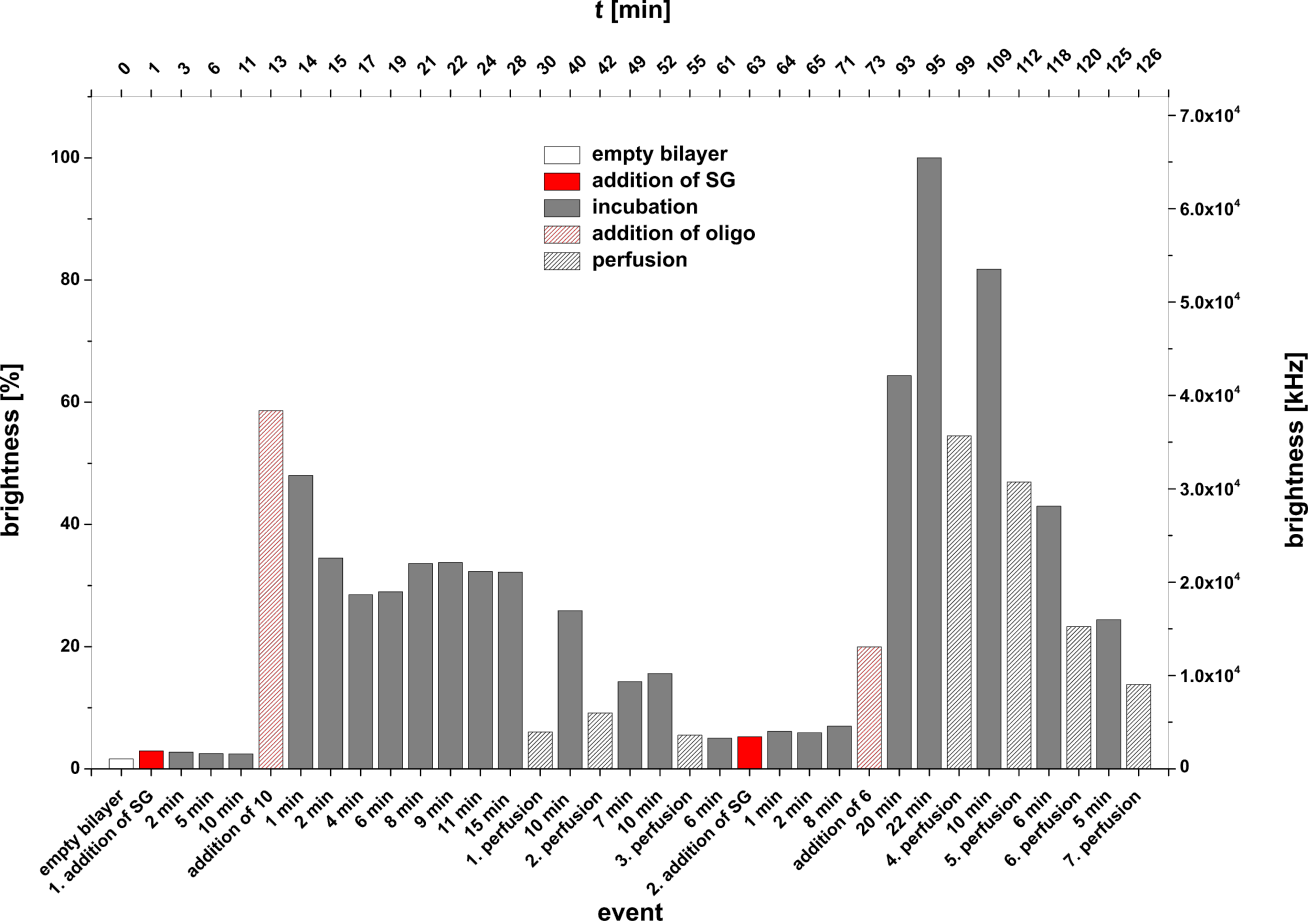
Experiment E in reversed order of component addition; **SG** + **10** + **6**.

In a further experiment using the lipo-oligonucleotide **10** we changed the order of the addition of the three components of the ternary complex to the *cis* compartment of the bilayer slide (Figure 13). Within the first 11 min we studied the interaction between Sybr Green I and the empty bilayer. As can be seen no ponderable brightness of the bilayer could be detected after addition of the dye. However, after addition of the 24-mer **10** (at *t* = 13 min) the brightness increased to about 60% (normalized brightness, 100% ≈6.5 × 10^4^ kHz) and stays almost constant (35–50%) until *t* = 28 min indicating a strong interaction between the dye and the single stranded 24-mer **10** [20]. Such an interaction could not be observed between the dye and the duplex of the 12-mers **4** and **7**. A subsequent perfusion reduces the brightness significantly to 5%. Interestingly, a subsequent waiting period of 10 min leads again to an increase of the bilayer brightness back to about 25%. This is probably due to an additional delivery of the complex **10**·**SG** from the *cis* compartment or from the *cis* side of the hydrophobic Teflon-made annulus into the lipid bilayer.

**Figure 14:**
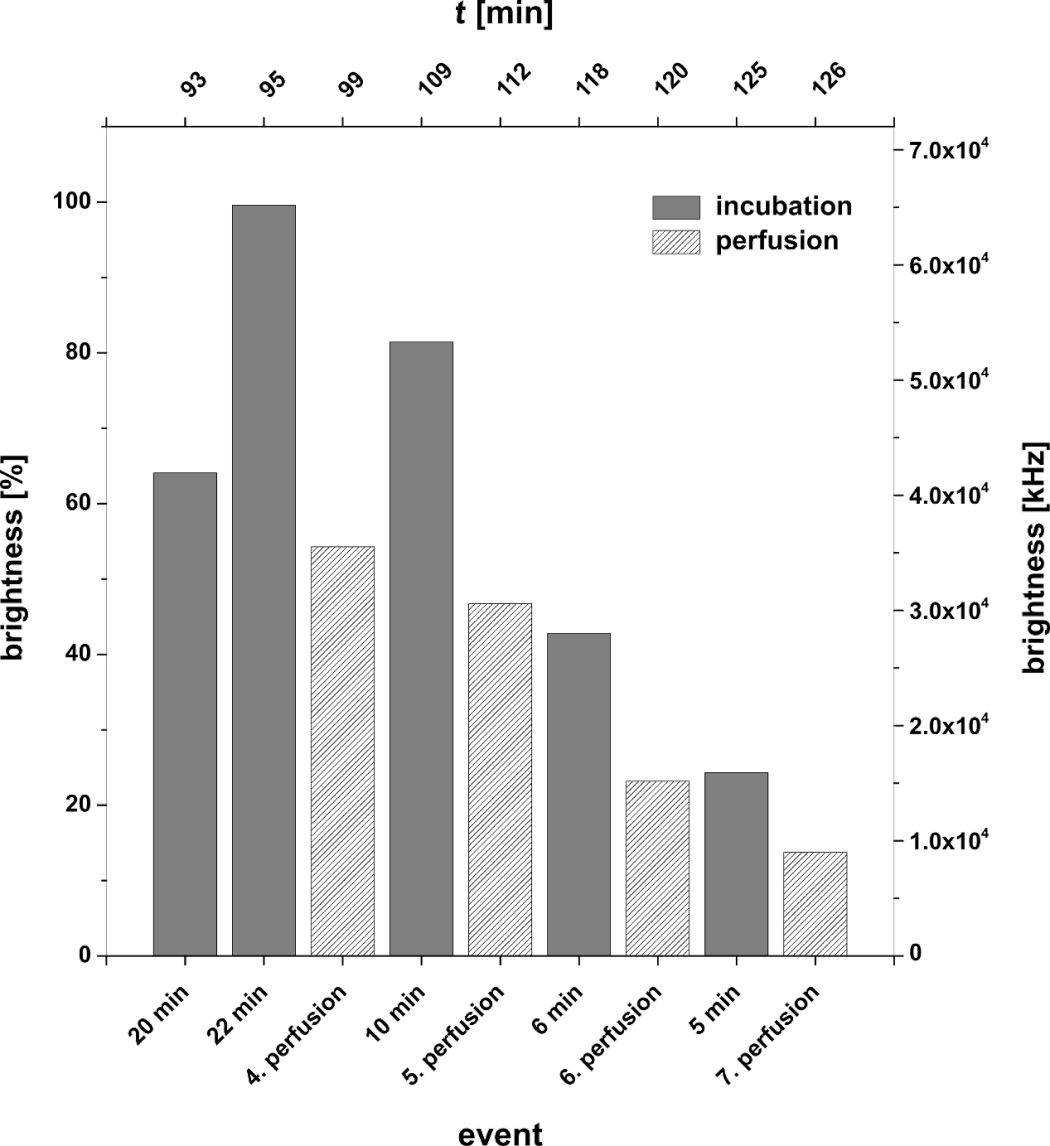
Bilayer brightness as a function of perfusion number and incubation periods for the experiment E in reversed order of component addition (**SG** + **10** + **6**).

At *t* = 63 min a second portion of the dye solution and at *t* = 73 min the oligomer **6**, being partly complementary to the lipo-oligonucleotide **10**, were added. Within an incubation time of 22 min after addition of **6** the full bilayer brightness (100 % at *t* = 95 min) was reached. Subsequent perfusion steps (1–4) interrupted by several incubation periods reduced the bilayer brightness from 100 to 10% (Figure 14). These results clearly indicate a significantly higher stability of the ternary complex at the bilayer compared to the complex of experiment D.

**Figure 15:**
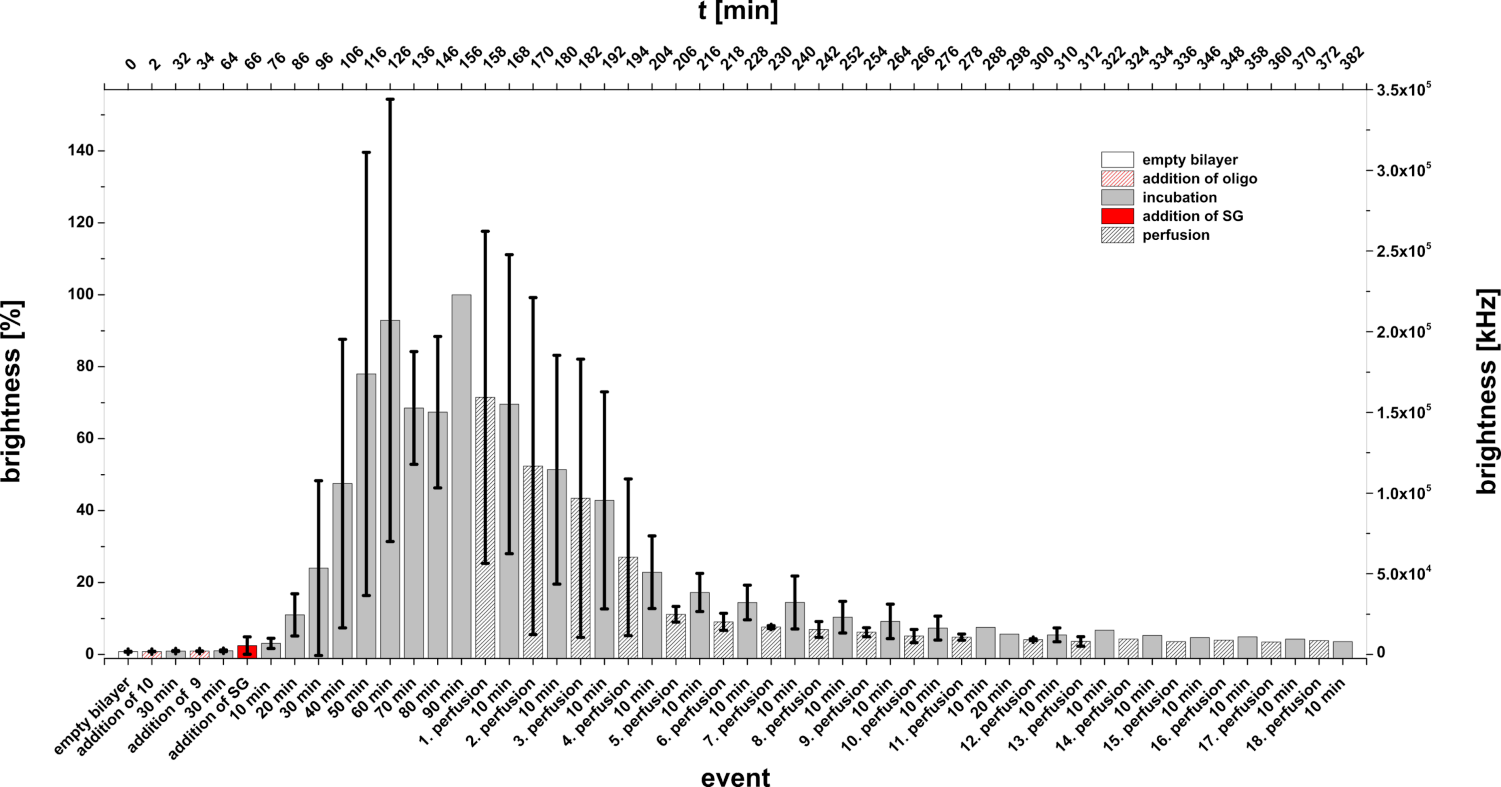
Bilayer brightness as a function of perfusion number and incubation periods for the experiment F (**SG** + **10** + **6**).

Experiment F: (**10** + **9** + **SG**). In the following the lipo-oligonucleotide **10** (24-mer) was immobilized on the lipid bilayer. After an incubation time of 30 min, an equimolar amount of the oligomer **9** – an oligomer of the same length and a central recognition sequence of 12 bases – was added, followed by addition of Sybr Green I after a further waiting period (30 min). Figure 15 displays the full protocol of mean brightness values (both, in % and kHz) as a function of time or the various events (incubation periods or perfusion). After the addition of the dye as last component of the ternary complex at *t* = 66 min, a maximal brightness is slowly developed within about 2 h. In this case, however, the maximal brightness (normalized 100% ≈6 × 10^4^ kHz) does not reach the value which had been observed in the experiments A, B, and E. Only in case of experiment D the maximal brightness is even lower than in case F (2.5 × 10^4^ kHz).

18 Subsequent perfusions, interrupted by waiting periods of 10 min each, gave after 382 min a bilayer brightness of ≈5% (= 1 × 10^4^ kHz). This indicates that the formation of the ternary complex **10**·**9**·**SG** occurs slowly, but when it is once formed, it remains stable for more than 6 h.

## Conclusion

**Figure 16:**
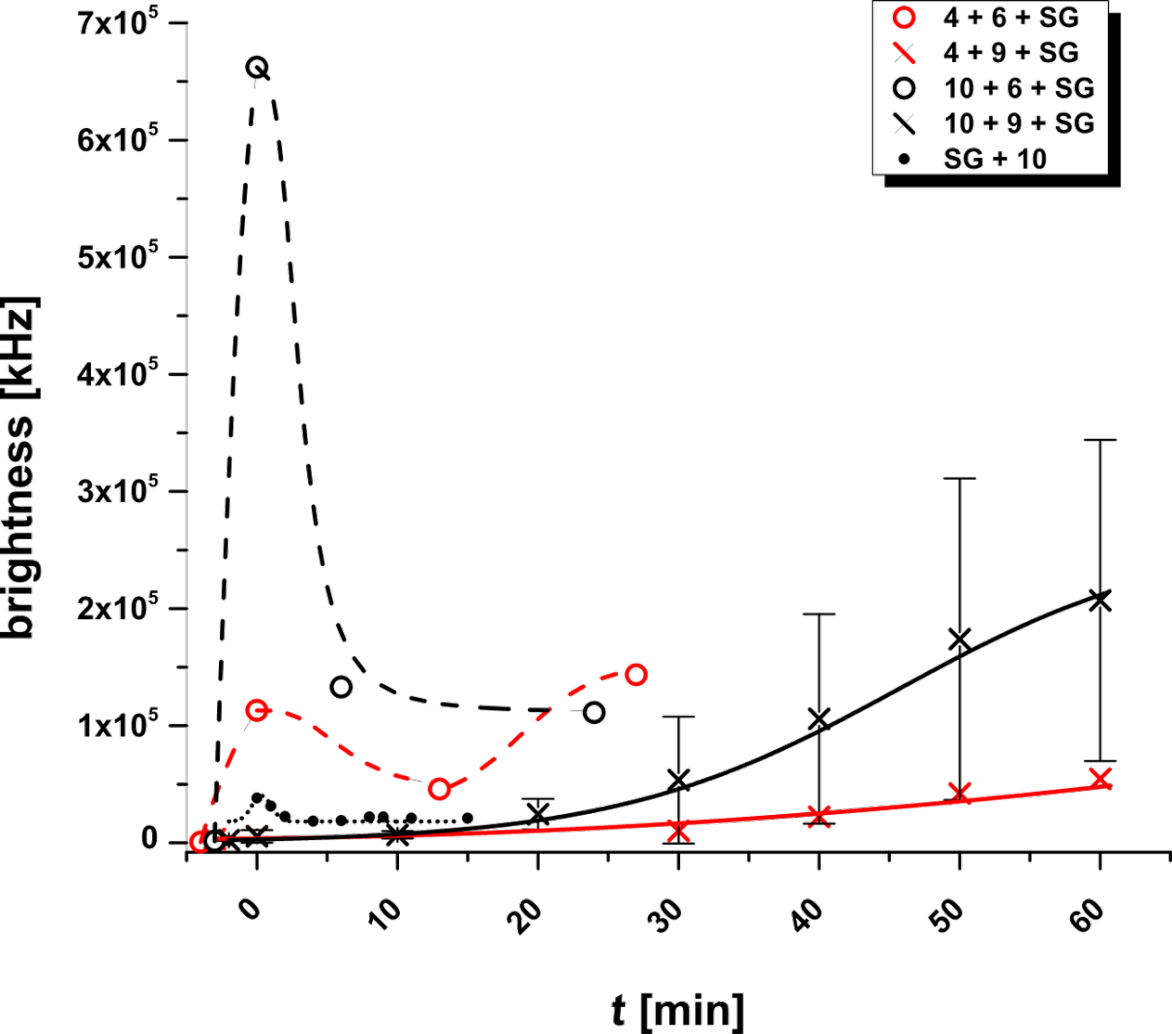
Kinetics of the tertiary complex formations of various lipo-DNA/DNA with Sybr Green I during incubation time *t* [min] after addition of the dye at *t*_0_ until the first perfusion.

Figure 16 shows a comparison of the kinetics of complex formation at the lipid bilayer surface for 5 experiments within the first 60 min (brightness [kHz] vs time [min]).

As can be seen, the fastest ternary complex formation occurs for **10** + **6** + **SG** (Experiment E) where the complex formation occurs at a distance of a hexamer spacer between the bilayer surface and the DNA duplex region with intercalated Sybr Green I. It can also be observed that, in this case, a maximal brightness at ≈6.5 × 10^5^ kHz is reached.

If the spacer is missing, the complex (**4** + **6** + **SG**, Experiment B) formation occurs significantly slower; the maximal brightness is reached at 1.5 × 10^5^ kHz. The same results are found in case of experiment A (**4** + **5** + **SG**) with two different fluorescent dyes – intercalating Sybr Green I and pending cyanine-5 (Figure 5).

For the scenario of experiment F (Figure 2) the complex formation kinetics drops even further, but the brightness reaches a plateau at ≈2 × 10^5^ kHz. The by far slowest complex formation is observed in case of experiment D (Figure 1) in which a steric clash between the ternary complex and the bilayer surface occurs. Here, no plateau value of the bilayer brightness is reached, and the complex proved to be highly labile.

Further control experiments such as 1. **6** + **7** (non-complementary strands + **SG**); 2. **5** + **7** (non-complementary strands) + **SG**; 3. **6** (lipophilized 12-mer) + **SG**; 4. empty bilayer + **SG** do not result in fluorescent bilayers. However, addition of Sybr Green I followed by the 18-mer **10** (or in the reversed order) to the *cis* chamber of the bilayer slide gives almost spontaneously a fluorescent bilayer with brightness values of ≈5 × 10^4^ kHz.

Further experiments concerning a multiple compartment chamber with a common aqueous sub-phase, as well as a thermostated device for a suppression of nonspecific base pairing are underway [15]. Moreover, ab initio molecular-dynamics- (AIMD)- and ab initio Monte-Carlo- (AIMC)- calculations of lipid–nucleic acid complexes with MC and MD simulations of semi-quantitative, “course-grained” models (CGM) are going to be performed (Prof. Dr. Philipp Maass, Department of Statistical Physics, University of Osnabrück).

**Figure 17:**
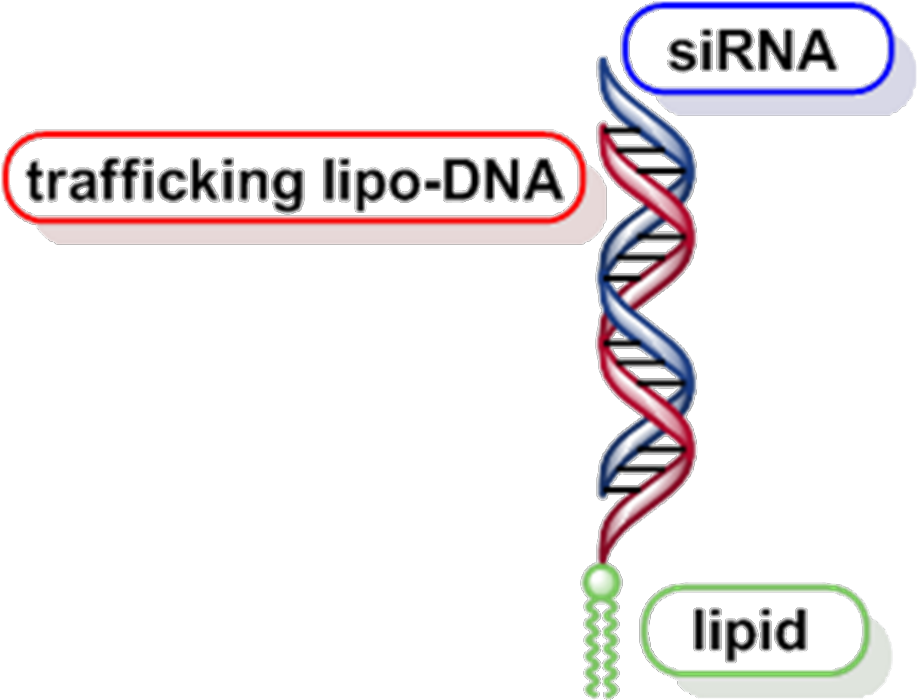
Trafficking of a siRNA by a lipophilized DNA.

Studies described in this manuscript as well as of those of a forthcoming paper which deals with the interaction of a defined oligonucleotide single strand, carrying nucleolipid head groups of different lipophilicity with lipid bilayer membranes, might be of importance for the optimization of the in vivo delivery of lipophilic siRNA [13] (e.g., by DNA trafficking as shown in Figure 17), as well as of lipid derivatives of nucleoside antimetabolites [23–24]. Moreover, studies of a transdermal application of lipo-oligonucleotides through the human Stratum corneum by iontophoresis techniques are underway.

## Experimental

### Materials

1-Palmitoyl-2-oleyl-*sn*-glycero-3-phosphoethanolamine (POPE) and 1-palmitoyl-2-oleyl-*sn*-glycero-3-phosphocholine (POPC) were purchased from Avanti Polar Lipids (Alabaster, Al), *n*-decane from Alfa Aesar (Karlsruhe, Germany), dimethyl sulfoxide (DMSO), potassium chloride (KCl), 3-morpholinopropane-1-sulfonic acid (MOPS) and 2-amino-2-hydroxymethylpropane-1,3-diol (TRIS) were purchased from Roth (Karlsruhe, Germany).

All oligonucleotides **4–10** (Figure 1) were synthesized, purified and characterized by MALDI–TOF mass spectrometry by Eurogentec SA (Liege, Belgium). In each case the detected mass confirmed the corresponding calculated mass. MALDI–TOF–MS (*m*/*z*): 4247.7 (**4**, [M + H]^+^; calcd 4247.4); 4178.4 (**5**, [M + H]^+^; calcd 4178.1); 3645.5 (**6**, [M + H]^+^; calcd 3644.5); 4248.1(**7**, [M + H]^+^; calcd 4247.4); 3643.2 (**8**, [M + H]^+^; calcd 3644.46); 7306.4 (**9**, [M + H]^+^; calcd 7312.9); 6076.4 (**10**, [M + H]^+^; calcd 6077.6).

**4** 5’-d(**1a**-p-TAG GTC AAT ACT)-3’

**5** 5’-d[(Cy5)-p-AGT ATT GAC CTA]-3’

**6** 5’-d(AGT ATT GAC CTA)-3’

**7** 5’-d(**1a**-p-ATC CAG TTA TGA)-3’

**8** 5’-d(TCA ATT GAC GAT)-3’

**9** 5’-d(TTT TAT AGT ATT GAC CTA TAT TTT)-3’

**10** 5’-d(**1a**-p-TTT TAT TAG GTC AAT ACT)-3’

We have chosen the particular sequences 5’-d(TAG GTC AAT ACT)-3’ and 3’-d(ATC CAG TTA TGA)-5’, because they do neither form hair pins nor self-complementary duplexes. The lipophilized strand and the complementary oligomer contain the same number of all canonical nucleotides, so that a mixture of 1 A_260_ units, each of both strands gives an equimolar mixture. For nearly all base pairs nearly all nearest neighbour combinations exist. Both strands have the same composition; the unmodified duplex exhibits a melting point of 46 °C in a phosphate buffer solution.

A solution of Sybr Green I in DMSO with undisclosed concentration was purchased from Applichem (Darmstadt, Germany) and used as delivered. The concentration of the dye solution (≈1 µg/mL) was measured UV–vis spectrophotometrically using the published extinction coefficient of Sybr Green I at λ_max_ of 494 nm (73.000 M^−1^cm^−1^) [20–21].

### Methods

**General.** A double-beam spectrophotometer (Specord 205, Analytik Jena GmbH, Jena, Germany) was used for spectrophotometric measurements. The UV–vis absorption spectra were carried out using ultra-micro quartz cuvettes type 105.202-QS from Hellma Analytics (Müllheim, Germany).

**Figure 18:**
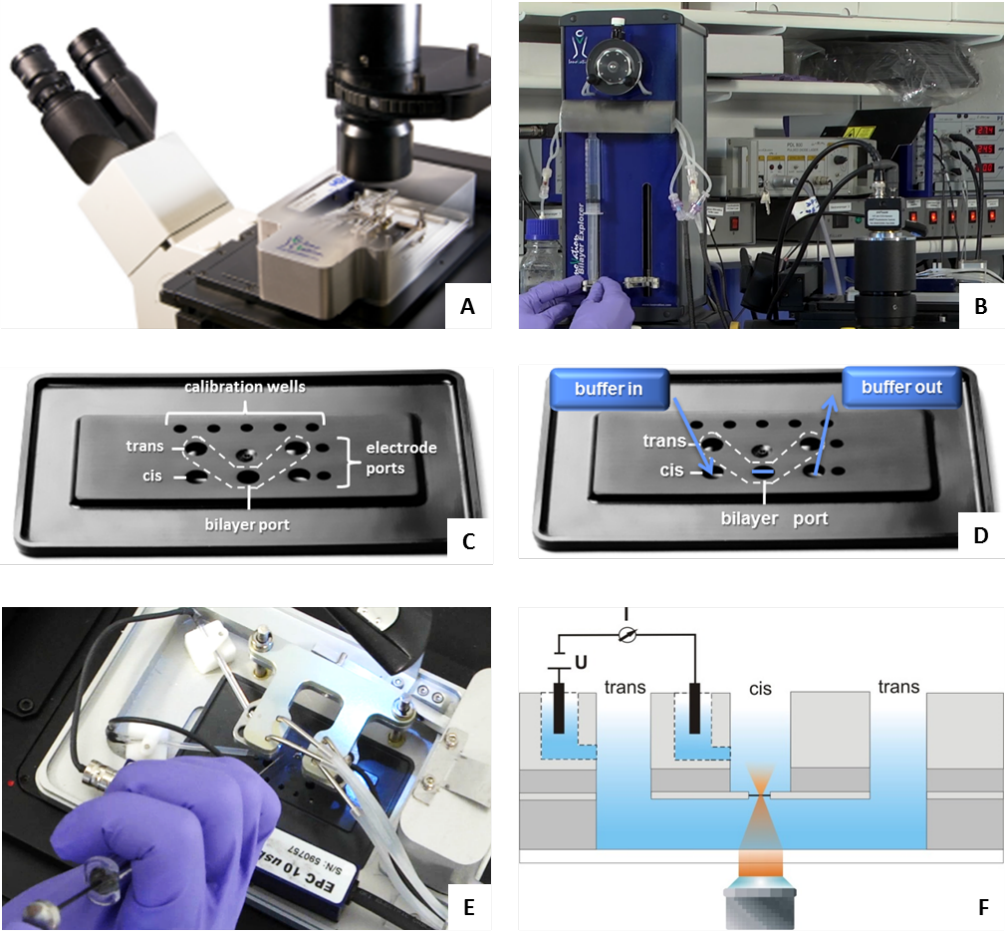
A) Stage unit of the ‘Ionovation Explorer’ mounted on a standard inverted fluorescence microscope. B) The perfusion unit (on the left) during filling the syringes with buffer. C) Labelling of the single compartments and ports of the ‘Bilayer Slide’. These are disposable, optically transparent microfluidic sample carriers with perfusion capabilities. D) Schematic demonstration of the *cis* chamber perfusion. E) Shot of the device from above during addition of the lipid mixture into the bilayer port of the slide. On the left, electrodes are visible, connected with the amplifier; on the right hand side tubes connected with the perfusion unit can be seen. F) Vertical cut of a buffer-filled bilayer slide, demonstrating its general design. The bilayer slide encloses two microfluidic channels (*cis* and *trans*) which are separated by a thin medical-grade PTFE (= polytetrafluoroethylene, Teflon) foil. This foil hosts a central 100 µm aperture which is located 120 µm above the cover slip and thus within the working distance of high NA (= numerical aperture) objectives. It is the only connection between the *trans* and the *cis* channels. When a lipid solution is painted across the aperture, a bilayer is formed spontaneously. The electrodes in the *ci*s and *trans* channels allow an online monitoring of the bilayer integrity, as well as electro-physiological recordings.

**Bilayer fabrication and incorporation of lipo-oligonucleotides therein.** Similar as described in [19], in the following the automated bilayer fabrication is described. Horizontal bilayers were fabricated automatically using a lipid mixture of 1-palmitoyl-2-oleyl-*sn*-glycero-3-phosphoethanolamine (POPE) and 1-palmitoyl-2-oleyl-*sn*-glycero-3-phosphocholine (POPC) (8:2, w/w, 10 mg/mL of *n*-decane) within the “Bilayer Slides” (Figure 18C,D) and an add-on for the inverted confocal microscope (Bilayer Slides and Ionovation Explorer, Ionovation GmbH, Osnabrück, Germany). After pre-filling with buffer (250 mM KCl, 10 mM MOPS/Tris, pH 7), the slide was inserted into the stage unit mounted on an inverted confocal microscope (Figure 18A). Ag/AgCl electrodes were mounted and after the addition of 0.2 µL of POPE/POPC lipid to the *cis* compartment using a 1 µL bended Hamilton syringe (CH-Bonaduz) (Figure 18E), the automated bilayer production was started; a modified painting technique, in which the air-water interface paints the lipid across the aperture was applied. The bilayer formation was monitored optically and electrically.

As soon as a stable bilayer has been established (C > 50 pF) the lipophilized oligonucleotide solution (8 µL, 500 nM) was injected into the *cis* compartment of the “Bilayer Slide”. During the incubation time of 30–40 min the bilayer integrity was monitored by continuous capacitance measurements. After the incubation of the first sample the second sample of a complementary or non-complementary oligonucleotide was injected into the *cis* compartment and incubated for the next 30 minutes. Subsequently, the Sybr Green I solution (8 µL, undiluted) was injected and incubated until the highest brightness amount was reached. In all graphs the brightness was given either in kHz or it was normalized (0–100%). In the meantime every 10 minutes a z-scan was performed (see below). After the incubation steps the *cis* compartment was perfused repeatedly for 30 s (1.1 mL/min, each), and the bilayer was inspected by a confocal fluorescence microscope. Each perfusion step using the Ionovation perfusion unit (Figure 18B) was followed by an incubation step of 10 minutes. This procedure was repeated until the brightness of the bilayer finished decreasing continuously and turned out to be stable.

A confocal laser scanning microscope (Insight Cell 3D, Evotec Technologies GmbH, Hamburg, Germany) equipped with a 488 nm argon-ion laser, (Multiline, 2014-25MLYVW (max. total power 50 mW) from JDS Uniphase Corporation (Milpitas, CA,USA), as well as with a 635 nm emitting laser diode (LDH-P-635, PicoQuant, D-Berlin) und 470 nm (P-C-470B, PicoQuant*,* Berlin, Germany), a 40× water-immersion objective (UApo 340, 40×, NA = 1.15, Olympus, Tokyo, Japan), and an Avalanche photodiode detector (SPCM-AQR-13-FC, Perkin-Elmer Optoelectronics, Fremont, CA, USA) were used for the optical measurements. Fluorescence irradiation was obtained with an excitation laser power of 60 ± 5 µW (for 488 nm), 200 ± 20 µW (635 nm) and 50 ± 5 µW (470 nm) right in front of the objective. Z-scans were performed by scanning the confocal laser spot in XY direction with a rotating beam scanner and movement of the objective in Z direction. The movement in all directions is piezo-controlled, which allows a nanometer precise positioning.

In summary, each measuring protocol was carried out as follows: (I) a reference scan of the stable pure bilayer was performed; (II) then the lipophilized oligonucleotide sample was added and incubated for 30 min, (III) this step was followed by the addition of the complementary and non-complementary sample, respectively, and further incubation of 30 min; (IV) finally the cyanine-dye Sybr Green I solution (DMSO) was added to allow the quantification of double stranded DNA, and the solution was incubated until the brightness in the bilayer reached the highest amount; (V) afterwards additional scan series were performed after each perfusion of the *cis* compartment (for 30 s; 1.1 mL/min) and (VI) followed by an incubation of 10 min.

In order to analyze the 2D images they were previously edited with Image JA 1.44 and the obtained data was evaluated with OriginPro 8 (OriginLab Corporation). For an evaluation of the z-scan data, the brightness within the bilayer was measured; the mean area of the bilayer cross section amounts to 353 ± 19 counts per pixel (N = 201). Next, the brightness was determined by summing up the number of pixels. 1 Pixel equals 1 kHz or 1 µm.

All devices (Figure 18) as well as the general techniques used in this paper have been described in detail in a preceding manuscript [19].
